# Optimization of Extraction of Bioactive Compounds from *Baphicacanthus cusia* Leaves by Hydrophobic Deep Eutectic Solvents

**DOI:** 10.3390/molecules26061729

**Published:** 2021-03-19

**Authors:** Zhiqin Xu, Yini Cai, Qing Ma, Zhimin Zhao, Depo Yang, Xinjun Xu

**Affiliations:** 1 School of Pharmaceutical Sciences, Sun Yat-Sen University, Guangzhou 510006, China; xuzhq3427@163.com (Z.X.); caiyn5@mail2.sysu.edu.cn (Y.C.); zhaozhm2@mail.sysu.edu.cn (Z.Z.); lssydp@mail.sysu.edu.cn (D.Y.); 2 China Resources Sanjiu Medical & Pharmaceutical Co., Ltd., Shenzhen 518110, China; maq@999.com.cn

**Keywords:** *Baphicacanthus cusia* leaves, bioactive compounds, ultrasound-assisted extraction, response surface methodology

## Abstract

Deep eutectic solvents (DESs) are considered as efficient and green solvents for the extraction of bioactive compounds from medicinal plants. In this work, a novel method of DES-based ultrasound-assisted extraction of bioactive compounds from *Baphicacanthus cusia* leaves (BCL) was established. Systematic screening and the morphology of the original and treated BCL were observed with scanning electron microscopy to determine the extraction efficiency of different solvents. The extraction conditions were optimized by Box–Behnken design (BBD) tests and the optimal extraction conditions were as follows: lactic acid/L-menthol ratio of 5: 2 (mol/mol), solid–liquid ratio of 80.0 mL/g and temperature of 60.5 °C. The extraction yields of tryptanthrin, indigo and indirubin reached 0.356, 1.744 and 0.562 mg/g, respectively. The results of a 2,2-diphenyl-1-picrylhydrazy (DPPH) radical scavenging activity test indicated the feasibility of DESs in the extraction of bioactive compounds. This study indicated that L-menthol/lactic acid was a green and efficient solvent for the extraction of bioactive compounds from BCL, and DES-based ultrasound-assisted extraction could be used as an effective application strategy for the extraction of bioactive compounds from medicinal plants.

## 1. Introduction

**Figure 1:**
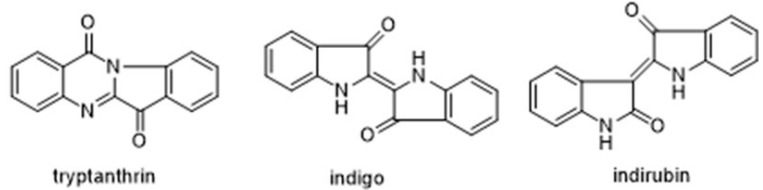
Chemical structures of tryptanthrin, indigo and indirubin.

*Baphicacanthus cusia* (Nees) Bremek is an herbal plant of the family *Acanthaceae*, mainly distributed in southern China, India and Myanmar. It is widely used as folk and clinical medicine [1,2,3]. The roots, stems and leaves of *Baphicacanthus cusia* can all be used as medicine and are commonly used in antiviral therapy. The roots of *Baphicacanthus cusia* are used as medicine named “Nan-Ban-Lan-Gen” in Chinese, and the leaves and stems are processed and used as medicine named “Qing-Dai” in Chinese [3]. In recent years, Qing-Dai has been reported to possess various biological activities, such as antibacterial [4], and as therapy for chronic myelogenous leukemia [5], ulcerative colitis [6] and psoriasis [7]. Tryptanthrin, indirubin and indigo (Figure 1) are the main chemical constituents in *Baphicacanthus cusia* leaves (BCL), which display diverse activities. The latest research showed that the methanol extract and tryptanthrin from BCL exhibited strong antiviral activity against HCoV-NL63. Because HCoV-NL63 is similar to SARS-CoV and COVID-19 in highly conserved sequence and structure, tryptanthrin may be developed as one of the coronavirus drugs [8]. Besides, tryptanthrin also has antibacterial [9], anti-tumor [10], anti-inflammatory [11] and antioxidant activity [12]. Indirubin is a treatment for chronic myeloid leukemia and also has antiviral [13], anti-inflammatory [14] and antioxidant [15] effects. Indigo has been reported to have antibacterial [16], anti-inflammatory [6], antioxidant [6] and immunosuppressive effects [17]. Owing to the significant biological activities of these three compounds, it is necessary to develop a rapid and green extraction technology from BCL.

Green extraction technology needs to find green solvents that can replace the traditional toxic organic solvents. The extraction and purification of bioactive compounds from medicinal plants are generally performed with conventional organic solvents. According to reports, BCL usually was extracted with methanol [8,18], ethanol [1] and methanol–dichloromethane mixed solvent [19]. These solvents are volatile, toxic and environmentally unfriendly. Researchers have focused on developing green solvents with low toxicity and low cost in recent years. In 2003, Abbott et al. [20] first proposed the deep eutectic solvent (DES), which was a mixture of naturally occurring hydrogen bond donors (HBD) and hydrogen bond acceptors (HBA) at an appropriate molar ratio. DESs have the advantages of being easy to prepare, not volatile, biodegradable, low toxicity and low cost [21,22,23]. DESs have been used in various areas of the biotechnology and chemical industries. They were recognized as a promising solvent in extraction and separation processes [23]. Until now, DESs have been widely used in the extraction of bioactive compounds in Chinese herbal medicine, such as polyphenols [24,25,26], flavonoids [27,28] and alkaloids [29,30]. DESs are mostly hydrophilic, and hydrophobic DESs were first reported in 2015 [31]. Some new DESs that are based on the combination of menthol with alcohols and organic acids have been synthesized. These DESs showed a high degree of hydrophobicity, which indicated that menthol was a good candidate for the preparation of hydrophobic DESs [32,33]. Menthol can be extracted from plants in the genus *mentha* and has a wide range of sources. In addition, eutectic mixtures of menthol have been reported in the pharmaceutical field. For example, the eutectic mixture of menthol and borneol was used as a carrier for transdermal drug delivery [34].

As a new green technology, ultrasound has a wide range of applications in various fields. For example, ultrasound can assist water coagulation regeneration, and its operation is simple, it takes a short time, and offers environmental protection [35]. Ultrasound can assist in the synthesis of new chitin derivatives, and the combination with light radiation in heterogeneous selective catalysis is a very innovative method [36,37]. In terms of extraction and separation, ultrasonic treatment can extract plant proteins from different sources [38]. The bioactive compounds in medicinal plants are usually extracted by using solvent extraction, thermal reflux extraction, ultrasound-assisted extraction and microwave-assisted extraction technology. It was reported that the ultrasound-assisted extraction method had higher extraction yields for indirubin and indigo than solvent extraction and thermal reflux extraction methods [39]. Compared with other modern extraction methods, the ultrasound-assisted extraction method has the advantages of shorter extraction period, lower temperature, being simpler, more environmental protection and higher yield. 

Response surface methodologies (RSM) is an optimization method integrating experimental design and mathematical modeling, in which various factors and their interactions influence the response. This method is widely used in chemical, agricultural, pharmaceutical, environmental and mechanical engineering fields. Box–Behnken design (BBD) is simpler, easier to arrange and more efficient than other designs [40]. It was reported that Zhao et al. used RSM to optimize the extraction conditions of indirubin and indigo from *Isatis indigotica* Fort [15]. 

In this paper, an ultrasound-assisted extraction method based on hydrophobic DESs is reported for the extraction of three bioactive compounds (tryptanthrin, indigo and indirubin) from BCL. In the experiment, a total of six different DESs were prepared, and the extraction results were comprehensively studied. The HBD–HBA molar ratio, solid–liquid ratio and temperature were investigated by RSM. The extraction yields of three bioactive compounds were taken as the response value to determine the optimal extraction process parameters. In addition, 2,2-diphenyl-1-picrylhydrazy (DPPH) assay was used to evaluate the antioxidant activities of DES extracts from BCL.

## 2. Results and Discussion

### 2.1. Evaluation of DES Extraction Efficiency

**Figure 2:**
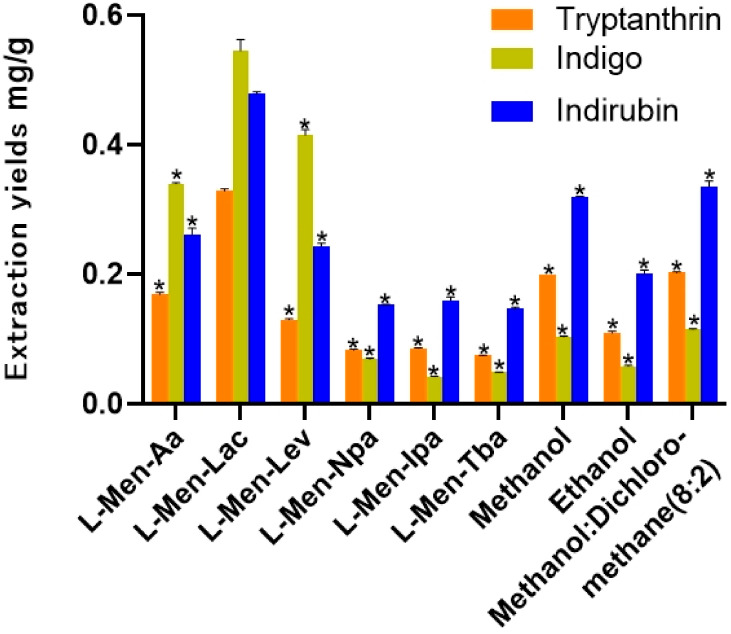
Extraction yields of tryptanthrin, indigo and indirubin using different DESs and traditional solvents [1,18,19]. Extraction yields of three active compounds with the above solvents were significantly different from those of L-men-lac, which were indicated by asterisk * (*p* < 0.05). Data were analyzed by the Student’s *t*-test.

It is particularly important to correctly select the DES type to extract the target compound from the sample. The preliminary experiments found that hydrophilic DESs with choline chloride and betaine as HBA have low extraction yields of three bioactive compounds of BCL. So the extraction of the three target compounds was suitable for hydrophobic DES extraction. Following methods used in previous reports [32,33], we selected six organic acids and alcohols that can form eutectic mixtures with L-menthol as components of DESs. These compounds include acetic acid, lactic acid, levulinic acid, *n*-propanol, isopropanol and *tert*-butanol, as shown in Table 1. In the screening experiment, in order to reveal the effect of DESs, under the same ultrasound-assisted conditions (ultrasonic temperature 35 °C ultrasonic extraction time 30 min, solid–liquid ratio 20 mL/g), tryptanthrin, indirubin and indigo were extracted from BCL by using various kinds of DESs and traditional solvents, respectively. The results are summarized in Figure 2.

As can be seen from Figure 1, of the conventional organic solvents of methanol, ethanol and methanol: dichloromethane (8:2), methanol: dichloromethane (8:2) extracted the tryptanthrin (0.2023 mg/g), indigo (0.1148 mg/g) and indirubin (0.3356 mg/g) from BCL with a higher yield. It is worth noting that the extraction yields of organic acid-based DESs (L-Men-Aa, L-Men-Lac, L-Men-Lev) were much higher than these of alcohol-based DESs (L-Men-Npa, L-Men-Ipa, L-Men-Tba) for tryptanthrin, indigo and indirubin, which might be related to their strong acidity. That indicated the organic acids as HBD could increase the solubility of alkaloids. The extraction yields of organic acid-based DESs for three bioactive compounds were higher than or close to these of traditional solvents, while the extraction yields of alcohol-based DESs for the three bioactive compounds were lower than these of methanol: dichloromethane (8:2). The results showed that L-Men-Lac was the best extraction solvent with extraction yields of 0.3286 mg/g (tryptanthrin), 0.5451 mg/g (indigo) and 0.4782 mg/g (indirubin), respectively, which were higher than those of other DESs and traditional solvents. In DESs, the type of HBD determined the extraction efficiency of alkaloids. Using organic acid as an HBD can improve extraction yields, while alcohol acting as an HBD could show the effect of reducing extraction yields.

**Figure 3:**
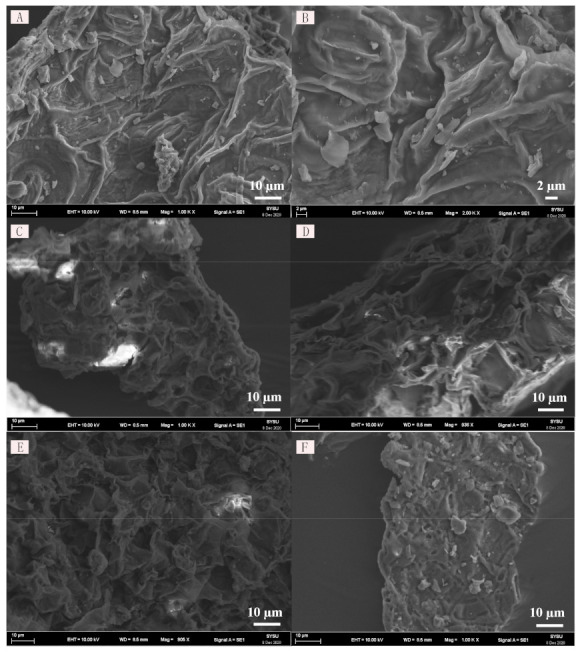
SEM images of *Baphicacanthus cusia* leaf (BCL) powder (**A**–**B**) before ultrasonic extraction and after ultrasonic extraction in (**C**) methanol, (**D**) methanol: dichloromethane (8:2), (**E**) L-Men-Lac and (**F**) L-Men-Tba baths.

In order to determine the influence of DESs on the samples, scanning electron microscopy (SEM) was used to observe the morphology of the samples of BCL before and after treatment in this paper, as shown in Figure 3. As shown in Figure 3A,B, the cell wall surfaces of the powders were smooth and neat, and the adjacent pores of the cell walls were closely arranged. In contrast, the cell wall surfaces became rougher after extraction with ethanol, methanol-dichloromethane (8:2), L-Men-Lac and L-Men-Tba. The structures of cell walls were destroyed and pores appeared. This was due to damage to cells and cell walls by ultrasonic waves, thereby exposing the target alkaloids to the extract. After extraction by L-Men-Lac Figure 3E, a large number of voids appeared and the adjacent pores were loosely arranged. Nevertheless, some minor structural damage and a few pores appeared on the surface of the sample after extraction by L-Men-Tba Figure 3F. That was consistent with the extraction yields reported in Figure 2.

The polarities of the three bioactive compounds in the sample are relatively weak, so they are suitable for the use of hydrophobic DESs. By changing the molar ratio of the components, the physical and chemical properties of DESs can be changed [41]. Adjusting the appropriate molar ratio and under the action of ultrasound, the three active compounds can be better extracted into DESs and the components with large polarity difference can be reduced. In addition, DESs probably could dissolve and hydrolyze cellulose, resulting in destruction of the cell walls [30]. Thus, higher amounts of the target compounds were exposed to the extraction solution. The results showed that L-Men-Lac was suitable for the extraction of tryptanthrin, indigo and indirubin from BCL. According to reports in the literature [33,42,43], the ultrasound-assisted extraction method based on DES can also be used to extract representative phytochemical constituents from *Ginkgo biloba* leaves, including flavonoid glycosides, terpenoids and flavonoids, phytocannabinoids from the *Cannabis sativa* plant and lycopene from the by-product of tomato processing. Combined with experimental results and literature, L-Men-Lac was used for subsequent experiments.

### 2.2. Fourier-Transform Infrared Spectroscopy (FT-IR) Analysis of L-Men-Lac

**Figure 4:**
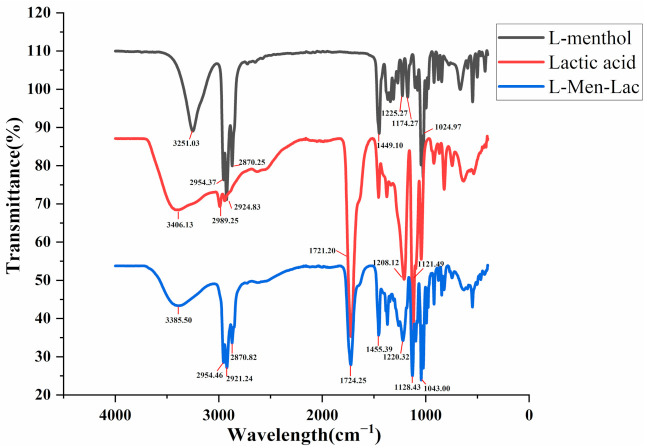
FT-IR spectra of L-menthol, lactic acid and L-Men-Lac.

Recently, DES has been widely used in the extraction of natural products as an environmental-friendly solvent. L-menthol was used as starting HBA to synthesize hydrophobic DESs with different organic acids or alcohols for the extraction of natural products with weak polarity [42,44]. In hydrophilic DESs, one is a hydrogen bond donor and the other is a hydrogen bond acceptor. The establishment of hydrogen bonds between two compounds is responsible for the formation of eutectic mixtures. By comparing the FT-IR spectra of L-menthol, lactic acid and the optimal solvent (L-Men-Lac), the overlapping infrared spectra of L-menthol, lactic acid and DES were obtained, as shown in Figure 4. An inspection of FT-IR spectra of the L-Men-Lac revealed a shift of the O–H band of L-menthol from 3251.03 to 3385.50 cm^−1^. The carbonyl band from the lactic acid was originally located at low wave number values (1721.20 cm^−1^) and it broadened and shifted to the highest values (1724.25 cm^−1^) in the L-Men-Lac. A similar phenomenon has been reported for DESs consisting of menthol and carboxylic acids or alcohols [32,33,38].

### 2.3. Optimization of the Extraction Parameters by RSM

**Figure 5:**
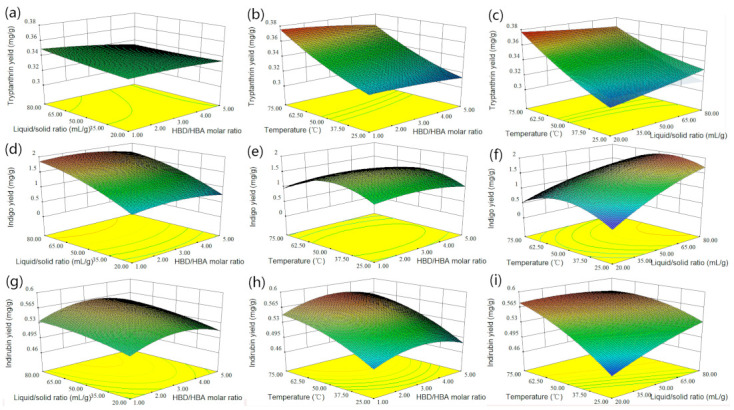
Response surfaces for the interactions of independent variables on (**a**–**c**) tryptanthrin yield, (**d**–**f**) indigo yield and (**g**–**i**) indirubin yield.

RSM can reveal the relationship between the response and the conditions that affect the response. After the preliminary test, the optimized extraction parameters were determined as liquid-to-solid ratio, temperature and HBD–HBA molar ratio. The yields of the three bioactive compounds were taken as the response values and a 17-run BBD with three variables and three levels (Table 2 and Table 3) was used to fit a first-order response surface. Three dimensional (3D) response surface analyses were performed on the multiple nonlinear regression models of the three bioactive compounds to describe the interaction of operational parameters (Figure 5). The experimental data were simulated by the Design Expert 8.0 Software package and the extraction yields of the target compounds could be predicted by the second-order polynomial equations, as follows:

Y_1_ = 0.271 + 3.913 × 10^−3^A + 6.597 × 10^−4^B + 8.357 × 10^−4^C − 5 × 10^−5^AB − 2.5 × 10^−5^AC − 1.033 × 10^−5^BC − 4.125 × 10^−4^A^2^ + 9.444 × 10^−7^B^2^ + 8.16 × 10^−6^C^2^ (1)

Y_2_ = −2.056 + 0.191A + 0.044B + 0.079C − 3.5 × 10^−4^AB − 1.2 × 10^−4^AC − 2.093 × 10^−4^BC − 0.033A^2^ − 1.624 × 10^−4^B^2^ − 7.203 × 10^−4^C^2^ (2)

Y_3_ = −2.056 + 0.191A + 0.044B + 0.079C − 3.5 × 10^−4^AB − 1.2 × 10^−4^AC − 2.093 × 10^−4^BC − 0.033A^2^ − 1.624 × 10^−4^B^2^ − 7.203 × 10^−4^C^2^ (3)

in which Y_1_, Y_2_ and Y_3_ were the extraction yields of tryptanthrin, indigo and indirubin (mg/g); A, B and C were the HBD–HBA molar ratio, solid–liquid ratio (mL/g) and temperature (°C).

The analysis of variance (ANOVA) was used to evaluate the optimal extraction conditions of L-Men-Lac for BCL and the relationship between the extraction yields of the three target compounds and variables. The results of ANOVA for the secondary model of tryptanthrin, indigo and indirubin are presented in Table 4. F-values and *p*-values can determine the significance of each coefficient. High F-values and small *p*-values mean that the corresponding variable is significant. The model F-values of tryptanthrin, indigo and indirubin were all more than 5.71 and the *p*-values were all less than 0.0016. That indicated the three models were significant. Meanwhile, “lack of fit” represents the part of the regression equation that fails to fit. ‘‘Lack of fit *p*-value’’ of more than 0.055 indicated that the “lack of fit” of the models was not significant and the experimental errors were small. In the three quadratic models, the liquid–solid ratio (B) and temperature (C) had greater effects than the HBD–HBA molar ratio (A).

Statistical analysis and three-dimensional (3D) RSM can illustrate the effects of independent variables and their interactions on the extraction yields of tryptanthrin, indigo and indirubin (Figure 5). Figure 5a,d,g shows 3D plots of the response surface for the extraction yields of tryptanthrin, indigo and indirubin, which are related to the HBD–HBA molar ratio (A) and liquid–solid ratio (B), respectively. It could be seen that the liquid–solid ratio (B) increased from 20 to 80 mL/g, which improved the extraction yields of the three target compounds. With the increase of the HBD–HBA molar ratio (A), the extraction yields of the target compounds increased. However, when the HBD–HBA molar ratio (A) exceeded a certain value, the extraction yields would decrease. That might be due to the increase of the viscosity of DESs with the increase of the molar number of lactic acid, which hindered the mass transfer efficiency of compounds from the plant matrix to the solvent. Liquid–solid ratio (B) played an important role in improving the extraction yields of the target compounds (Figure 5c,d,f,i). Increasing the liquid–solid ratio can significantly improve the extraction yields, which was related to increasing the contact area between powder and DES and improving the dissolution of the compounds. As shown in Figure 5b,e,h, temperature was an important factor affecting the extraction yields. With the increase of temperature from 25 to 75 °C, the extraction yields of tryptanthrin and indirubin increased. When the temperature rose to a certain level, the extraction yield of indigo decreased. High temperature could promote solute solubility and diffusivity in DES, but too high a temperature might change ultrasonic cavitation characteristics and strength of mass transfer [15].

With the extraction yields of tryptanthrin, indigo and indirubin as the response values, the optimal extraction conditions were as follows: lactic acid/L-menthol ratio of 5: 2 (mol/mol), solid–liquid ratio of 80.0 mL/g and temperature of 60.5 °C. Three parallel experiments were carried out under the optimal parameters. The extraction yields of tryptanthrin, indigo and indirubin were 0.356 ± 0.007, 1.744 ± 0.034 and 0.562 ± 0.007 mg/g, which were close to the predicted values (0.354, 1.727 and 0.566 mg/g) of the regression model, respectively. That verified the applicability of the model and the effectiveness of the optimal values within the range of extraction parameters.

Recovery of DESs and extracted compounds from DES extracts remains a challenge that has limited their large-scale application. At present, the reported recovery methods have mainly included macroporous resin adsorption [24], solid phase extraction [45] and liquid–liquid extraction [46]. However, the recovery rate of these methods was low, and DESs might be diluted and difficult to recycle and reuse. HSCCC (High-speed countercurrent chromatography) has also been found to be useful for the recovery of extracted compounds and DESs [47]. Compared with other methods, this method has the advantages of high recovery rate, simple operation, strong separation capability and easy reuse of recovered solvents, which will facilitate the application of DES in the field of extraction and separation.

### 2.4. DPPH Radical Scavenging Activities of DES Extracts

**Figure 6:**
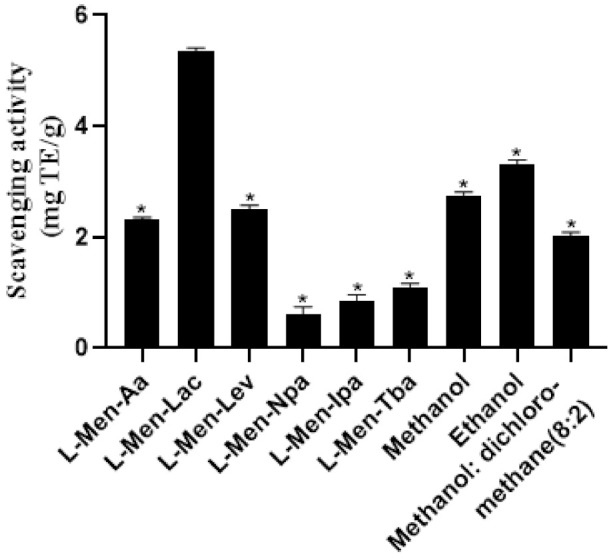
The free radical scavenging effects of DES extracts of BCL determined by DPPH assays. DPPH radical scavenging activities of extracts with the above solvents were significantly different from that of L-men-lac, which was indicated by asterisk * (*p* < 0.05). Data were analyzed by the Student’s *t*-test.

DPPH is widely used to determine the antioxidant capacity of biological samples, pure compounds and extracts in vitro. This method is simple to operate and can initially evaluate the antioxidant activity of the sample. The results of DPPH radical scavenging activities of extracts are shown in Figure 6. The results showed that the selected DES extracts had good scavenging activity of DPPH free radicals and L-Men-Lac extract had the best DPPH free radical scavenging effect (5.35 ± 0.06 mg TE/g), which was higher than those of traditional solvent extracts. The free radical scavenging activities of L-Men-Aa and L-Men-Lev extracts were comparable to those of traditional solvent extracts, while the free radical scavenging activity of the other three alcohol-based DES extracts were much lower than those of traditional solvent extracts. In conclusion, the results of the DPPH test were similar to the optimized results of extraction solvents. L-Men-Lac was the extract solvent with the best antioxidant activity and the antioxidant activities of different solvents were as following: organic acid-based DESs > traditional solvents > alcohol-based DESs.

## 3. Materials and Methods

### 3.1. Materials Reagents and Equipment

*Baphicacanthus cusia* leaves were collected from Guangdong province, China and dried in the sun. The leaves were ground by a disintegrator, then sample powder was passed through a 60 mesh sieve and samples were collected with a particle size of 60 mesh. The powder (60 mesh) was dried in a vacuum drying oven (under the presence of phosphorus pentoxide) to a constant weight and stored in a dryer before being used in subsequent experiments.

Tryptanthrin (≥98%) and indigo (≥98%) were purchased from Shanghai Yuanye Bio-Technology Co. Ltd. (Shanghai, China). Indirubin (≥95%), L-menthol (99.5%), lactic acid (85.0–90.0%), laevulinic acid (99.0%), propanol (99.5%) and DPPH (96.0%) were all purchased from Shanghai Macklin Biochemical Co. Ltd. (Shanghai, China). Acetic acid (≥99.5%), isopropyl alcohol (≥99.7%) and *n*-butanol (≥99.5%) were all purchased from Tianjin Jindongtianzheng Precision Chemical Reagent Factory (Tianjin, China). HPLC-grade acetonitrile was purchased from Sigma Aldrich (Saint Louis, USA).

The LC 2030 HPLC analysis system (Shimadzu, Tokyo, Japan) was equipped with a quaternary pump, a thermostatted column compartment and a DAD detector. The PS-60A ultrasonic water bath was obtained from Jiekang Ultrasonic Instrument Co. Ltd. (Dongguan, China). The DF-101S Collector Constant Temperature Heating Magnetic Stirrer was obtained from Gongyi Yuhua Instrument Co. Ltd. (Gongyi, China). The Spectrum 100 FT-IR was obtained from Perkin Elmer Inc (Waltham, MA, USA). The EVO MA10 SEM was obtained from Carl Zeiss (China) Co. Ltd. (Shanghai, China). The microplate reader was obtained from BMG Labtech Inc (Offenburg, Germany).

### 3.2. Preparation of DES

In this study, DESs were prepared by the method used in the previous reports [39]. L-menthol as hydrogen bond acceptor (HBA) and organic acids or alcohols as hydrogen bond donor (HBD) were mixed in different ratios, stirred at 80 °C until the formation of homogeneous and transparent liquid. In this study, six kinds of DESs were prepared. 

### 3.3. Extraction Procedure

In initial screening, accurately weighted 0.05 g *Baphicacanthus cusia* (Nees) Bremek leaf powders were added to 1 mL of DES in a 2 mL centrifuge tube. After vortexing, the mixture was put into an ultrasonic bath at 35 °C, 250 W power and 40 KHz frequency for 30 min, followed by centrifugation at 12,000 rpm for 10 min. Subsequently, the solution was diluted with methanol, then filtered through a 0.22 μm filter and quantified using HPLC analysis. Each experiment was performed three times. 

### 3.4. Morphological Characterization Observation of BCL by Scanning Electron Microscope (SEM)

SEM was used to observe the differences of BCL before and after extraction with different solvents. The dried BCL powders were fixed on the sample table with double-sided tape and gold-plated. The morphologies of BCL samples before and after extraction with different solvents were examined by SEM at an acceleration voltage of 10 kV and 1000 × amplification, respectively.

### 3.5. FT-IR Measurements 

The FT-IR spectra measurements were performed using a Spectrum 100 FT-IR (Perkin Elmer, MA, USA). The prepared DES and its compounds were directly used for measurement. Data related to the spectral region were recorded between 4000 and 400 cm^−1^ at room temperature.

### 3.6. Characterization and Quantification of Extracted Compounds

The contents of tryptanthrin, indigo and indirubin were determined by HPLC. Chromatographic analysis was performed on a Unitary C18 column (250 × 4.6 mm i.d., 5µm, Acchrom). The mobile phase consisted of water (phase A) and acetonitrile (phase B). The gradient program was as follows: 0–15 min, 35%–56% B, 15–20 min, 56% B, 20–23 min, 95% B, 23–33 min, 95% B. The flow rate was 1.0 mL/min, the running temperature was at 35 °C, the injection volume was 10 μL and the wavelengths were 250 nm (for tryptanthrin) and 289 nm (for indigo and indirubin).

Calibration curves were established for tryptanthrin, indigo and indirubin by plotting concentrations of standard solutions and peak areas. The linear ranges, linear regression equations and related details are listed in Table S1 in Supporting Information.

### 3.7. Determination of Antioxidant Capacity

2,2-Diphenyl-1-picrylhydrazyl (DPPH) radical scavenging assay was used to evaluate the antioxidant capacities of the samples.

The antioxidant capacity of BCL extract was determined by DPPH radical scavenging assay [28] with slight modifications. DPPH radical scavenging activities were evaluated by the use of a microplate. Briefly, the reaction mixture containing 190 µL DPPH (193.7 µM freshly made methanol solution) and 10 µL sample/blank solution in methanol was taken in a 96-well microplate and incubated at room temperature for 30 min. The absorbance was measured at 517 nm. The known trolox concentrations (0–1.5 mmol/L) were used for calibration with a good linear relationship (r = 0.9991). The assay was carried out in triplicate, and the results were expressed as mg of trolox equivalent (mg TE)/g DW of the sample.

## 4. Conclusions

In this study, the DES-based ultrasound-assisted extraction method was used to extract bioactive compounds (tryptanthrin, indigo and indirubin) from BCL. After screening of different DESs, L-Men-Lac was proven to be an effective solvent for the extraction of the three bioactive compounds from BCL, which was superior to conventional organic solvents. In addition, SEM images showed that the morphologies of BCL treated with L-Men-Lac were obviously changed, which indicated that L-Men-Lac was beneficial to the exposure of bioactive compounds in the extract. The optimal extraction conditions were as follows: lactic acid/L-menthol ratio of 5:2 (mol/mol), solid–liquid ratio of 80.0 mL/g and temperature of 60.5 °C. The extraction yields of tryptanthrin, indigo and indirubin reached 0.356, 1.744 and 0.562 mg/g, respectively. The DPPH radical scavenging activities of DES extracts were also determined, which proved the feasibility of DESs in extracting bioactive compounds from Chinese herbal medicine. These results indicate that DES can replace the traditional solvent as a green and efficient solvent for the extraction of bioactive compounds from BCL, and it is an effective application strategy for the extraction of bioactive compounds from medicinal plants.

## Figures and Tables

**Table 1:** Different compositions of deep eutectic solvents (DESs) studied in this work.

No.	Abbreviation	Type of HBA	Type of HBD	Molar Ratio	Appearance at Room Temperature
1	L-Men-Aa	L-Menthol	Acetic acid	1:1	Transparent liquid
2	L-Men-Lac	L-Menthol	Lactic acid	1:1	Transparent liquid
3	L-Men-Lev	L-Menthol	Levulinic acid	1:1	Transparent yellow liquid
4	L-Men-Npa	L-Menthol	*n*-Propanol	1:1	Transparent liquid
5	L-Men-Ipa	L-Menthol	Isopropanol	1:2	Transparent liquid
6	L-Men-Tba	L-Menthol	*tert*-Butanol	1:2	Transparent liquid

**Table 2:** Factors and levels of response surface analysis.

Levels	Independent Variables		
	A (HBD–HBA Molar Ratio)	B (Liquid–Solid Ratio, mL/g)	C (Temperature, ℃)
−1	1	20	25
0	3	50	50
1	5	80	75

**Table 3:** Results of the Box–Behnken design (BBD) for the extraction of tryptanthrin, indigo and indirubin.

Run	Factors			Extraction Yields		
	A (HBD–HBA Molar Ratio)	B (Liquid–Solid Ratio, mL/g)	C (Temperature, ℃)	Tryptanthrin (mg/g)	Indigo (mg/g)	Indirubin (mg/g)
1	3	50	50	0.344	1.727	0.571
2	3	20	25	0.305	0.542	0.460
3	3	80	75	0.371	1.078	0.571
4	3	50	50	0.334	1.720	0.551
5	5	50	75	0.364	0.912	0.569
6	1	50	25	0.320	1.038	0.470
7	3	20	75	0.379	0.588	0.585
8	5	80	50	0.330	1.710	0.536
9	5	20	50	0.333	0.622	0.495
10	1	50	75	0.370	0.935	0.528
11	3	50	50	0.340	1.466	0.544
12	3	50	50	0.344	1.446	0.558
13	1	20	50	0.342	0.818	0.518
14	5	50	25	0.319	1.039	0.503
15	3	50	50	0.337	1.458	0.549
16	3	80	25	0.328	1.660	0.522
17	1	80	50	0.351	1.990	0.553

**Table 4:** ANOVA statistics of the quadratic model for the extraction yields of tryptanthrin, indigo and indirubin.

Variables	Tryptanthrin	F-Value	*p*-Value	Indigo	F-Value	*p*-Value	Indirubin	F-Value	*p*-Value
	Mean Square			Mean Square			Mean Square		
Model	6.94 × 10^−4^	22.76	0.0002	0.35	13.97	0.0011	2.02 × 10^−^^3^	5.71	0.0158
*A*	1.71 × 10^−4^	5.61	0.0497	0.031	1.23	0.3039	1.45 × 10^−^^4^	0.41	0.5428
*B*	5.51 × 10^−^^5^	1.81	0.2208	1.87	74.27	<0.0001	1.92 × 10^−^^3^	5.44	0.0524
*C*	5.62 × 10^−^^3^	184.15	<0.0001	0.073	2.91	0.1317	0.011	31.43	0.0008
*AB*	3.60 × 10^−^^5^	1.18	0.3133	1.76 × 10^−^^3^	0.070	0.7989	9.00 × 10^−^^6^	0.025	0.8777
*AC*	6.25 × 10^−^^6^	0.20	0.6645	1.44 × 10^−4^	5.72 × 10^−^^3^	0.9418	1.60 × 10^−^^5^	0.045	0.8375
*BC*	2.40 × 10^−4^	7.88	0.0263	0.099	3.92	0.0883	1.44 × 10^−^^3^	4.09	0.0829
*A^2^*	1.15 × 10^−^^5^	0.38	0.5593	0.074	2.92	0.1311	2.24 × 10^−^^3^	6.33	0.0400
*B^2^*	3.04 × 10^−^^6^	0.100	0.7614	0.090	3.57	0.1006	1.54 × 10^−^^4^	0.44	0.5300
*C^2^*	1.10 × 10^−4^	3.59	0.100	0.85	33.89	0.0006	8.31 × 10^−^^4^	2.35	0.1689
Lack of Fit	4.56 × 10^−^^5^	2.37	0.2111	0.030	1.41	0.3628	6.78 × 10^−^^4^	6.21	0.0551
*R^2^*	0.967			0.9473			0.8800		

## Data Availability

Data sharing not applicable.

## Supplementary Materials

The following are available online, Table S1: Calibration curves, linear ranges, LOD and LOQ for analytes by HPLC, Table S2: Extraction yields of three bioactive compounds from BCL using DESs and traditional solvents (values are expressed as mean ± D), Table S3: The free radical scavenging effect of DES extracts of BCL determined by DPPH assays, Figures S1–S9. HPLC chromatograms of BCL extracts.

## Sample Availability

Samples of the compounds are available from the authors.
