# Supplementary material for: Optimization of Extraction of Bioactive Compounds from Baphicacanthus cusia Leaves by Hydrophobic Deep Eutectic Solvents
Source: Molecules. 2021 Mar 19;26(6):1729. doi: 10.3390/molecules26061729 (PMC8003621; doi:10.3390/molecules26061729)
Supplement: Supplementary file 1 [file molecules-26-01729-s001.pdf]

Supporting information

Table S1. Calibration curves, linear ranges, LOD and LOQ for analytes by HPLC

No.	Wavelength (nm)	Calibration Curve	R^2	Linear Range ($\mu g \cdot mL^{-1}$)	LOD ($ng \cdot mL^{-1}$)	LOQ ($ng \cdot mL^{-1}$)
tryptanthrin	250	Y=143410X+1972.4	0.9998	0.792-19.796	15.837	47.510
indirubin	289	Y=87055X-5381.4	0.9998	0.985-24.624	29.549	98.496
Indigo	289	Y=104581X-84751	0.9992	0.918-45.903	55.084	183.612

LOD, limits of detection. LOQ, limits of quantification.

Table S2. Extraction yields of three bioactive compounds from BCL using DESs and traditional solvents (values are expressed as mean±SD)

Solvent	content（mg/g）		
	Tryptanthrin	Indigo	Indirubin
L-Men-Aa	0.1687±0.0032	0.3399±0.0016	0.2611±0.0100
L-Men-Lac	0.3286±0.0031	0.5451±0.0171	0.4782±0.0035
L-Men-Lev	0.1297±0.0015	0.4149±0.0083	0.2438±0.0042
L-Men-Npa	0.0827±0.0017	0.0686±0.0019	0.1526±0.0007
L-Men-Ipa	0.0857±0.0007	0.0419±0.0005	0.1598±0.0052
L-Men-Tba	0.0743±0.0004	0.0485±0.0004	0.1462±0.0026
Methanol	0.1997±0.0001	0.1035±0.0008	0.3196±0.0009
Ethanol	0.1092±0.0023	0.0580±0.0014	0.2015±0.0050
Methanol:Dichloromethane (8:2)	0.2023±0.0015	0.1148±0.0010	0.3356±0.0081

Table S3. The free radical scavenging effect of DESs extracts of BCL determined by DPPH assays

Solvent	mg TE/g
L-Men-Aa	2.33 ± 0.03
L-Men-Lac	5.35 ± 0.06
L-Men-Lev	2.49 ± 0.08
L-Men-Npa	0.63 ± 0.12
L-Men-Ipa	0.84 ± 0.12
L-Men-Tba	1.09 ± 0.07
Methanol	2.76 ± 0.06
Ethanol	3.30 ± 0.09
Methanol: Dichloromethane (8:2)	2.02 ± 0.07

Figure S1–S9. HPLC chromatograms of BCL extracts

Figure S1. HPLC chromatograms of BCL extracts using L-Men-Aa (wavelength=289 nm)

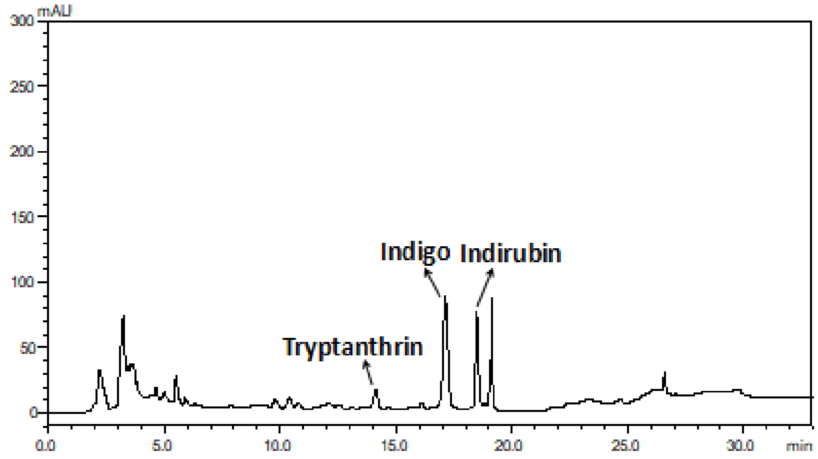

Figure S2. HPLC chromatograms of BCL extracts using L-Men-Lac (wavelength=289 nm)

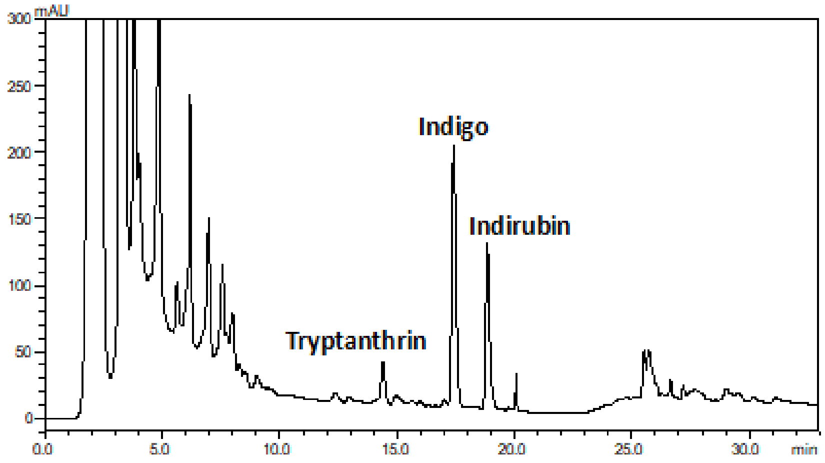

Figure S3. HPLC chromatograms of BCL extracts using L-Men-Lev (wavelength=289 nm)

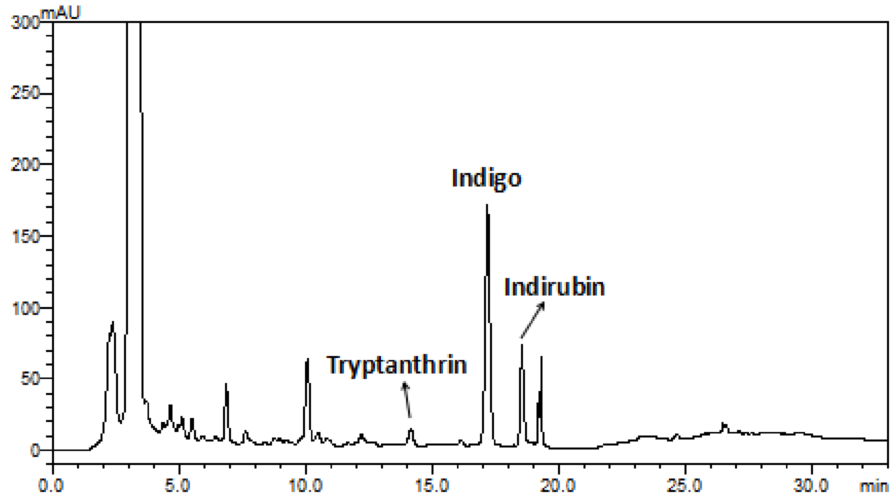

Figure S4. HPLC chromatograms of BCL extracts using L-Men- L-Men-Npa (wavelength=289 nm)

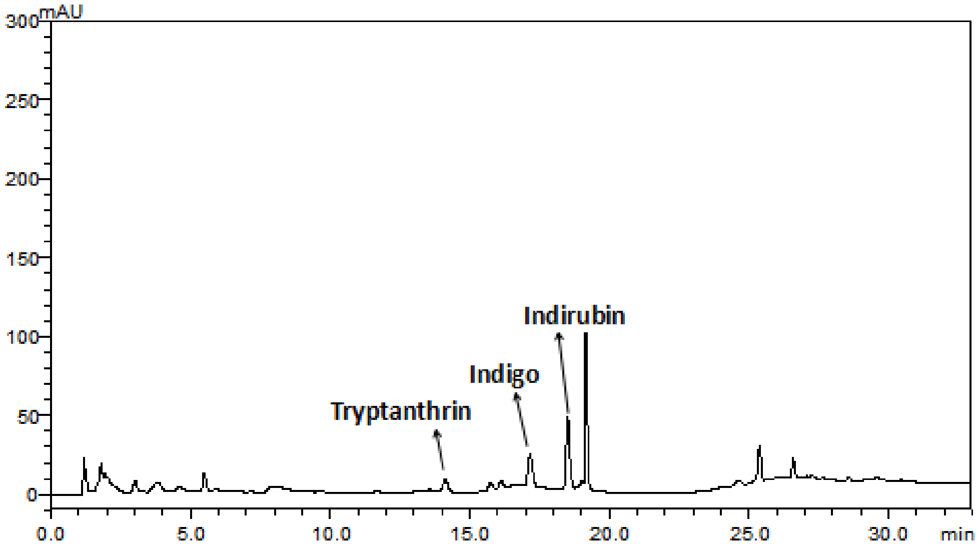

Figure S5. HPLC chromatograms of BCL extracts using L-Men- L-Men-Ipa (wavelength=289 nm)

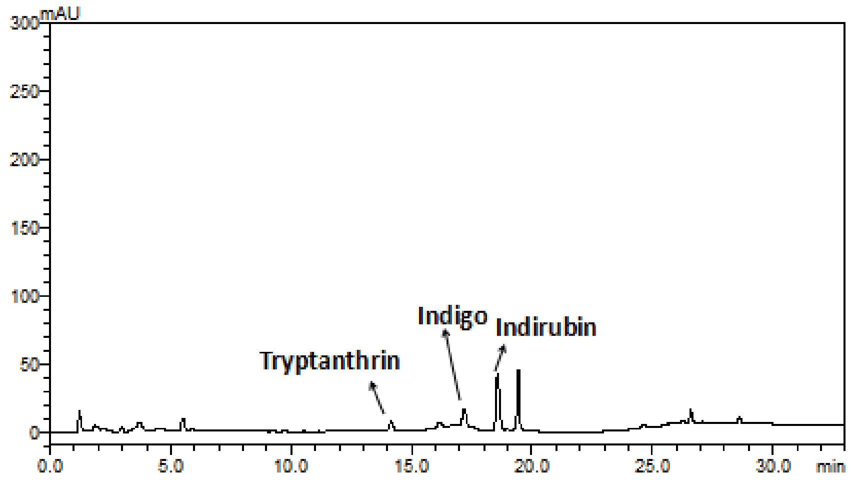

Figure S6. HPLC chromatograms of BCL extracts using L-Men-Tba (wavelength=289 nm)

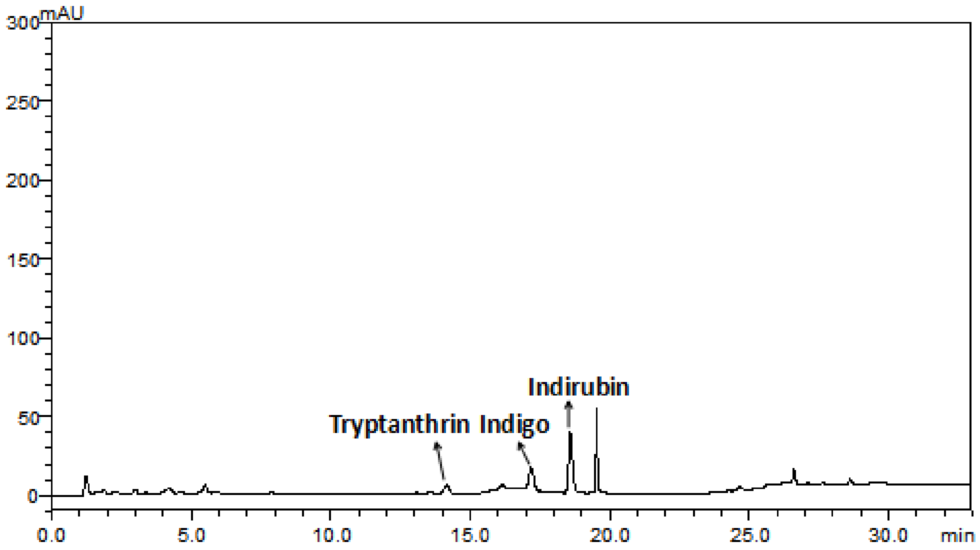

Figure S7. HPLC chromatograms of BCL extracts using ethanol (wavelength=289 nm)

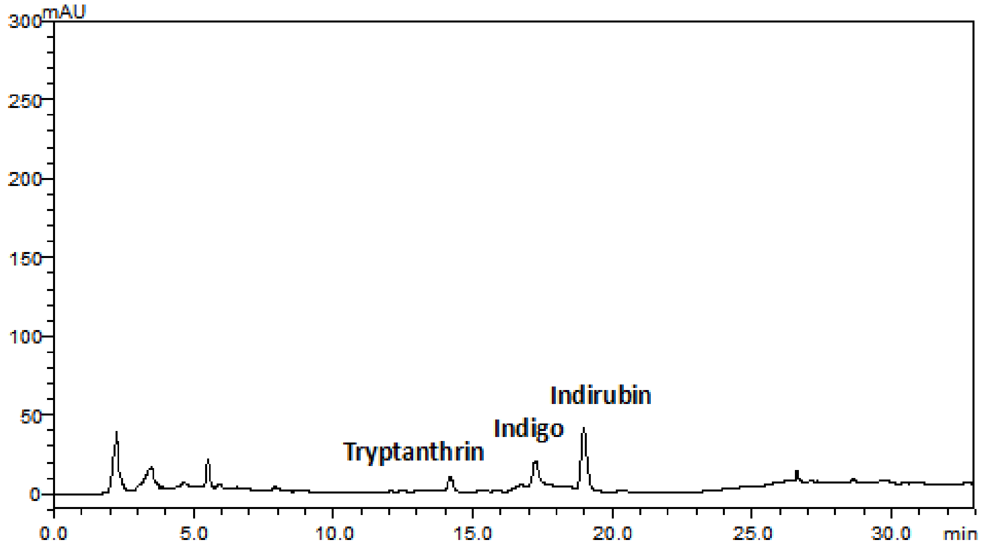

Figure S8. HPLC chromatograms of BCL extracts using methanol (wavelength=289 nm)

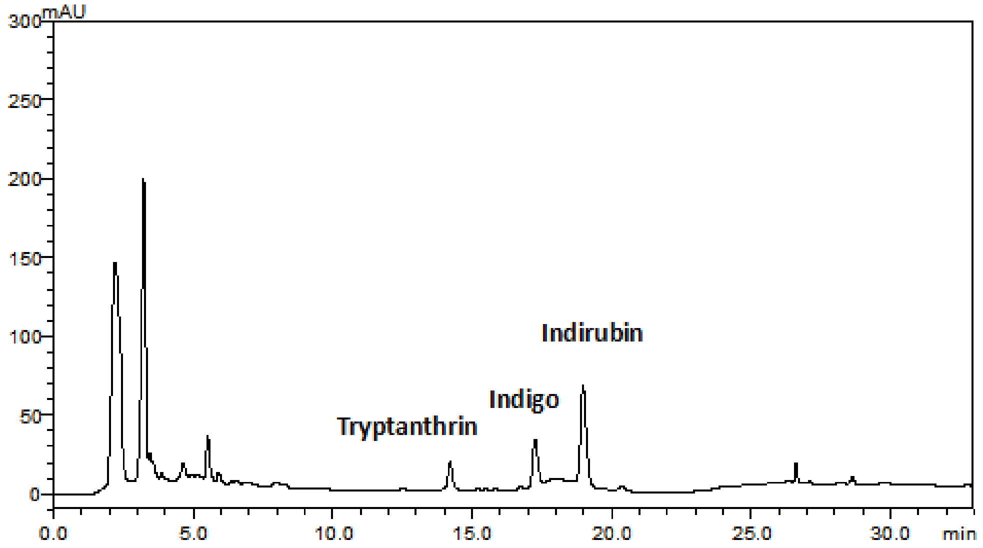

Figure S9. HPLC chromatograms of BCL extracts using methanol:dichloromethane (8:2) (wavelength=289 nm)

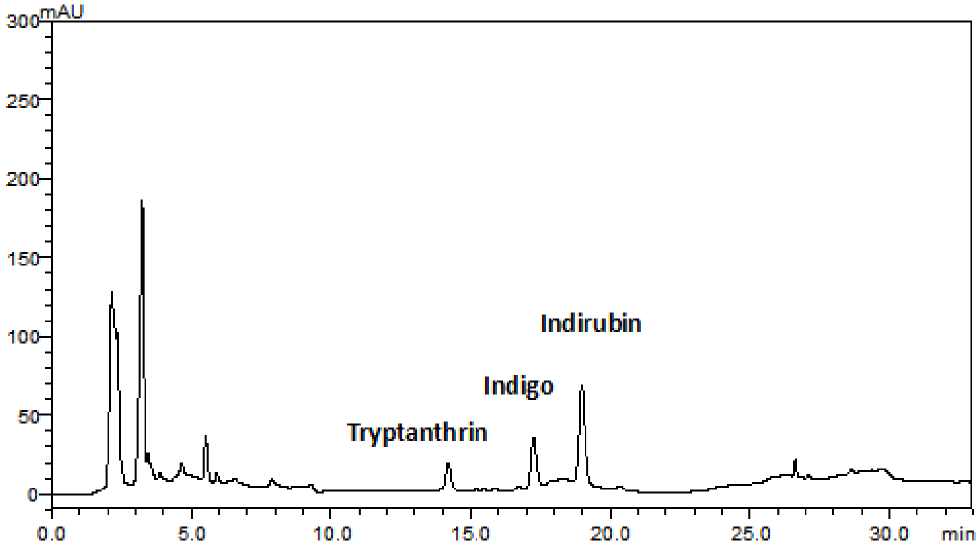

mAU
300
250
200
150
100
50
0
Indirubin
Indigo
Tryptanthrin
0.0
5.0
10.0
15.0
20.0
25.0
30.0
min
